# 2024 at *BMC Global and Public Health*: a year in review

**DOI:** 10.1186/s44263-024-00119-5

**Published:** 2024-12-23

**Authors:** Ben Cranfield, Gen Li, Gerrit John-Schuster

**Affiliations:** 1 Springer Nature, BioMed Central, London, UK; 2 Springer Nature, BioMed Central, Shanghai, China; 3 Springer Nature, BioMed Central, NY New York, USA

*An exciting year for BMC Global and Public Health comes to a close. With the support of our editorial board members and guest editors, we published impactful research and opinion and increased our recognition within the community.*

Building *BMC Global and Public Health* and expanding its reach and impact since its launch in 2023 has been an exciting and gratifying endeavor. After publishing 30 articles in 2023, we have seen a steady increase in recognition of the journal. Until now, we have published more than 80 research and opinion papers in 2024 and have launched more than 10 subject-specific collections led by guest editors.

All our collections cover timely topics and are aimed at addressing global health equity issues. A persistent challenge that faces global health is the delivery of integrated, coordinated, and continuous care, particularly in low- and middle-income countries (LMICs). In recognition of this fundamental inequity, we launched a collection called “Ensuring continuity of care in LMICs” (https://www.biomedcentral.com/collections/ELMIC) to promote the publication of highly impactful research in this area. Geography plays a key role in health care delivery, where the geographic setting in which people live can be a determining factor in how public health initiatives are designed and implemented. Our collection on “Geographic challenges to equitable healthcare” (https://www.biomedcentral.com/collections/GCEH) encourages submissions that focus on the intricacies between geography, geopolitics, topography, and global healthcare. Unjust healthcare is also evident in how vaccines are used internationally, where the recent pandemic has highlighted regional inequalities in vaccine research, development, and implementation. Therefore, our collection called “Ensuring equality in vaccine access: Addressing global inadequacies” (https://www.biomedcentral.com/collections/EEVA) aims to publish content that aims to reduce such variation in vaccine use.

To achieve health for all, the One Health approach, by recognizing the interconnectedness of humans, animals, and the environment, seeks to unite various sectors and disciplines to tackle issues ranging from biodiversity preservation to food security and infectious disease control. To advance our understanding of how best to foster policy and operational environments that support successful advances in addressing health challenges at the intersection between humans, animals, and the environment, we launched a collection that calls for submissions on intersectoral collaboration for One Health implementation (https://www.biomedcentral.com/collections/BDS).

In this year, we also published some exciting research that gains a lot of attention from the wider public health community. Systems approaches are crucial to solve complex real-world questions like public health challenges. In their study on the double burden of malnutrition, Quinteros-Reyes *et al.* demonstrated how community-based system dynamics modeling could be applied to the food system to understand the key drivers underlying the co-existence of overweight and stunting in Peru, with the participation of key stakeholders from different sectors [1]. Another study reported that systems modeling could be a useful tool to inform future investments to reduce suicidal thoughts and behaviors among young adolescents from LMICs [2], which is an emerging public mental health issue as supported by results from a cross-sectional survey of 45 LMICs also published in our journal [3].

In the post-COVID-19 era, it is important to consider how experiences during the pandemic shape population health behaviors in future public health emergencies. Using data from successive outbreaks of Ebola and COVID-19 in Liberia, it was found that people who had direct experience with the disease during the Ebola outbreak still adopted less precautionary behaviors during COVID-19, and their household members still had higher odds of testing positive for COVID-19 [4]. The result suggests that strategies to increase precautionary behavior among people with direct COVID-19 exposure should be carefully considered in future epidemics.

Obesity represents another form of epidemic that remains a global public health priority. Recent developments in weight loss medicines, particularly semaglutide, are a promising solution for overweight diabetic patients but also a temptation for non-diabetic people who want to lose weight. Given recently reported shortages in the supply of this drug, researchers have conducted a worldwide infodemiological study to understand people’s interest in semaglutide through patterns of online search behaviors [5]. Raubenheimer and colleagues were able to pinpoint specific social media events that led to increased international interest in semaglutide and highlighted that weight loss was a major theme, while diabetes was mostly absent or weak. We highlighted and discussed this work and its implications in more detail in a published blog post on the Springer Nature Research Communities pages (https://go.nature.com/4e4tNl0).

Of course, the above collections and articles highlighted are only a snapshot of the exciting and impactful content published at *BMC Global and Public Health* this year.

All this would not have been possible without a dedicated editorial team, including both professional in-house editors, academic editorial board members, and guest editors. Our editorial board has grown from an initial group of around 20 researchers to more than 40, and they dedicate their time to support the journal’s mission—providing a forum to promote health and well-being, advance health equity, and inform policy-making worldwide.

## Data Availability

Not applicable.

## References

[1] Quinteros-Reyes C, Seferidi P, Guzman-Abello L, et al. Mapping food system drivers of the double burden of malnutrition using community-based system dynamics: a case study in Peru. BMC Global Public Health. 2024;2:15. 10.1186/s44263-024-00045-6. 39681951 10.1186/s44263-024-00045-6 PMC11622957

[2] Ospina-Pinillos L, Skinner A, Sánchez-Nítola MN, et al. Systems modelling and simulation to guide targeted investments to reduce youth suicide and mental health problems in a low–middle-income country. BMC Global Public Health. 2024;2:70. 10.1186/s44263-024-00101-1. 39681918 10.1186/s44263-024-00101-1 PMC11622891

[3] Zhan Y, Wang P, Zhan Y, et al. Clustering of lifestyle risk factors in relation to suicidal thoughts and behaviors in young adolescents: a cross-national study of 45 low- and middle-income countries. BMC Global Public Health. 2024;2:24. 10.1186/s44263-024-00055-4. 39681898 10.1186/s44263-024-00055-4 PMC11622970

[4] Skrip LA, Weller MB, Dukuly S, et al. Unraveling the effects of the Ebola experience on behavior choices during COVID-19 in Liberia: a mixed-methods study across successive outbreaks. BMC Global Public Health. 2024;2:22. 10.1186/s44263-024-00054-5. 39681907 10.1186/s44263-024-00054-5 PMC11622887

[5] Raubenheimer JE, Myburgh PH, Bhagavathula AS. Sweetening the deal: an infodemiological study of worldwide interest in semaglutide using Google Trends extended for health application programming interface. BMC Global Public Health. 2024;2:63. 10.1186/s44263-024-00095-w. 39681910 10.1186/s44263-024-00095-w PMC11622965

